# Clinical Profile of Unilateral Proptosis in a Tertiary Care Centre

**DOI:** 10.1155/2017/8546458

**Published:** 2017-10-03

**Authors:** Susan Dsouza, Pooja Kandula, Gurudutt Kamath, Manjunath Kamath

**Affiliations:** Department of Ophthalmology, Kasturba Medical College, Manipal University, Mangalore, India

## Abstract

Proptosis, the forward protrusion of the eyeball, is a common manifestation of a wide variety of diseases inside the orbit and its spaces. Its diagnosis is usually a combined effort of the ophthalmologist, otolaryngologist, neurosurgeon, and radiologist. A clinical study of twenty-five cases with unilateral proptosis were studied in different age groups over a period of about 3 years under different headings like distribution, clinical features, radiological features, histopathological aspects, management, and outcomes of diseases. Proptosis measurement was done by simple/plastic ruler exophthalmometry, and diagnosis was made after a detailed clinical examination and ancillary tests. Treatment modality was decided based on radiological and histopathological examination reports, which included medical surgery, radiotherapy, and chemotherapy or a combination of all. In our study, most of the patients were in the age group of more than 60 years. The M : F ratio is 3 : 1. One case had a large proptosis of 18 mm above normal and 2 cases were as small as 3 mm. Diagnosis was mainly done by clinical features and confirmed by radiological and histopathological features. Most of them were treated medically (13 cases, i.e., 52%) and the rest by surgery with a combination of radiotherapy/chemotherapy (12 cases, i.e., 48%).

## 1. Introduction

Proptosis is described as an abnormal protrusion of the eyeball [1], and in relation to the skull, proptosis is measured from the corneal apex to the outer orbital margin of the orbit, with the eye looking straight [2].

The causes of unilateral proptosis are innumerable. The eye is a major crossroad for all the structures around it which help in its support and functioning, which when affected extends into the orbit causing proptosis. It can be the most dramatic of the orbital symptoms, especially if it has an acute onset.

A clear knowledge of the aetiologies will help the ophthalmologist to suspect, diagnose early, and provide treatment.

An attempt has been made to study the aetiology, clinical features, histopathology, and management of proptosis and its outcome.

## 2. Material and Methods

This is a retrospective record based on a clinical analysis of 25 cases of patients who presented to the eye outpatient department with unilateral proptosis during a period of 3 years. A detailed ophthalmological examination was done and a simple/plastic ruler was used to measure proptosis. Readings greater than 18 mm were considered as having proptosis [3]. A difference between both the eyes more than 2 mm was also considered significant.

Demographic details, clinical presentation, investigation details, and treatment details were noted. X-rays, B-scan, CT scan, and MRI orbit were performed to confirm the diagnosis.

## 3. Results

In our study of 25 patients presenting with unilateral proptosis, the age of the patients were between 6 and 75 years, and most of them were in the age group of more than 60 years old (24%) as shown in Table 1.

18 were males (72%) and 7 were females (28%).

The minimum proptosis was 3 mm and the maximum was 18 mm above the normal. Most of the patients had a right-sided proptosis, that is, 13 cases (52%). It was axial in 15 cases and eccentric in 10 cases as in Table 2.

Headache and protrusion of eye were the commonest presented complains in these patients along with other complains like pain, defective vision, epiphora, and diplopia shown in Table 3.

**Figure 1:**
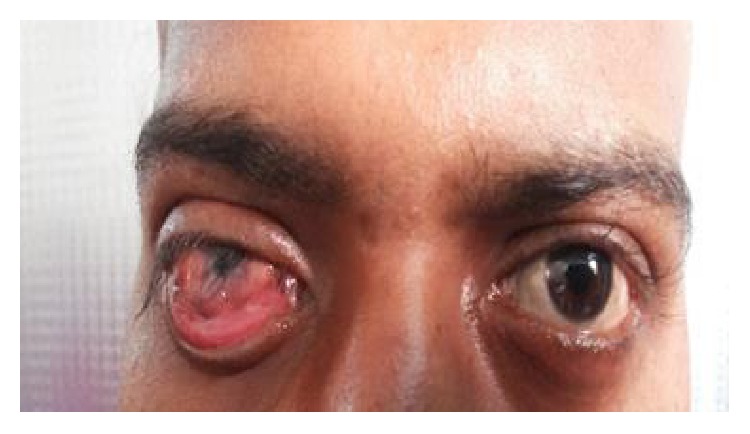
Pseudotumor—axial.

**Figure 2:**
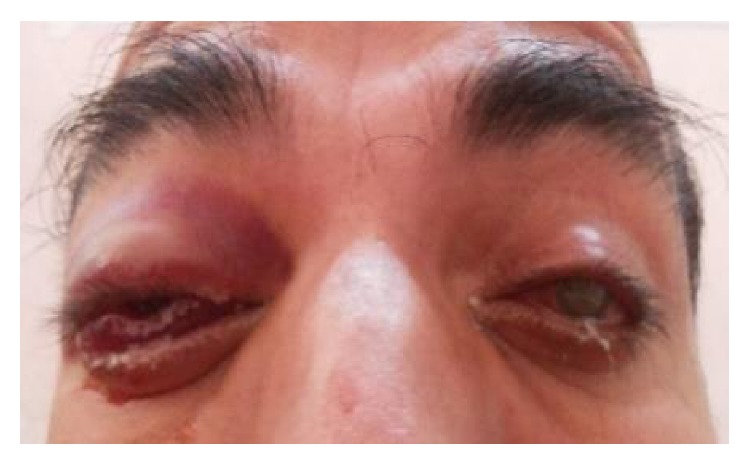
Retrobulbar haemorrhage—axial.

**Figure 3:**
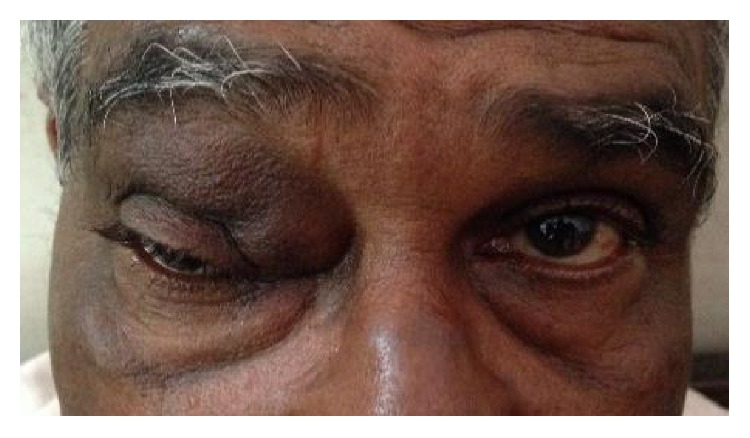
Angular dermoid—eccentric.

**Figure 4:**
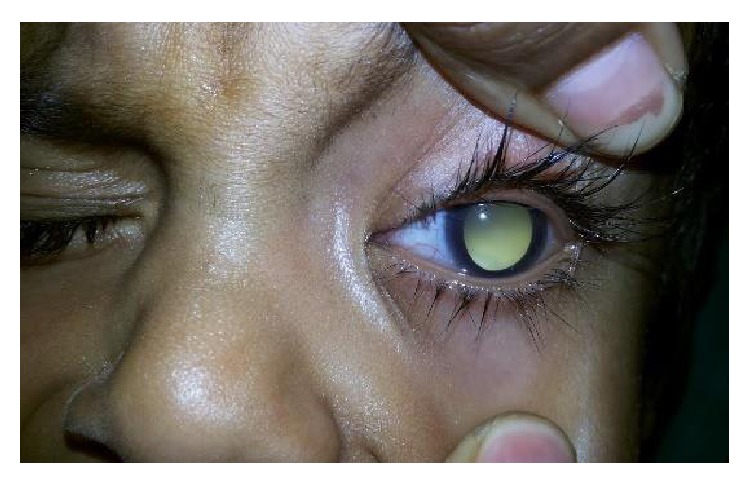
Retinoblastoma—axial.

**Figure 5:**
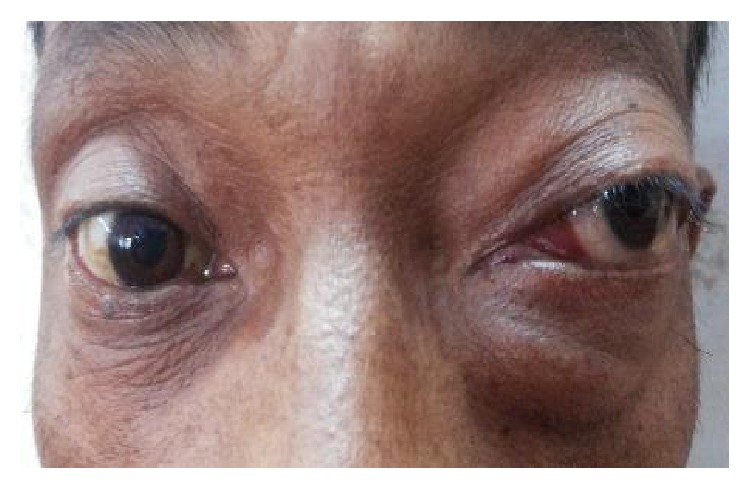
Squamous cell carcinoma—eccentric.

**Figure 6:**
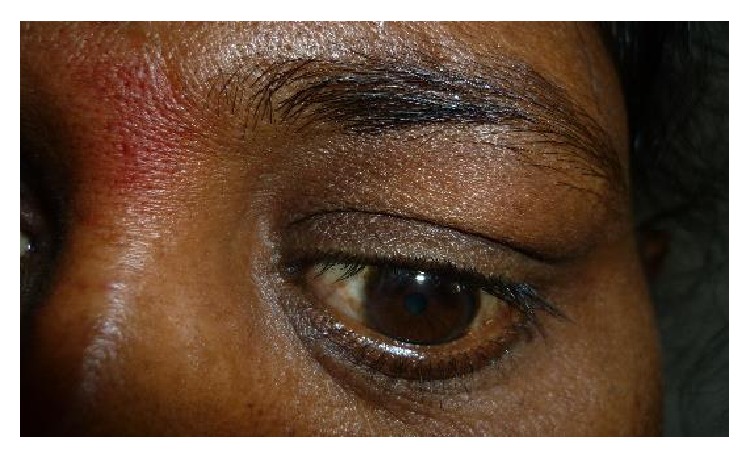
Pleomorphic adenoma—eccentric.

**Figure 7:**
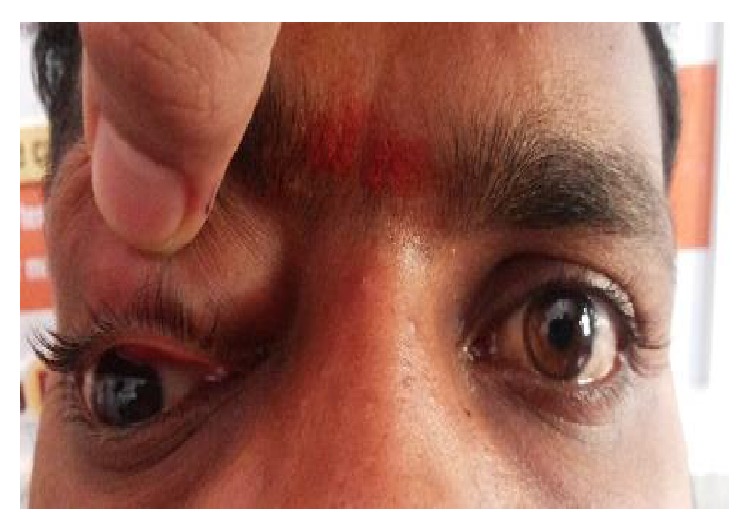
Osteoblastoma—eccentric.

**Figure 8:**
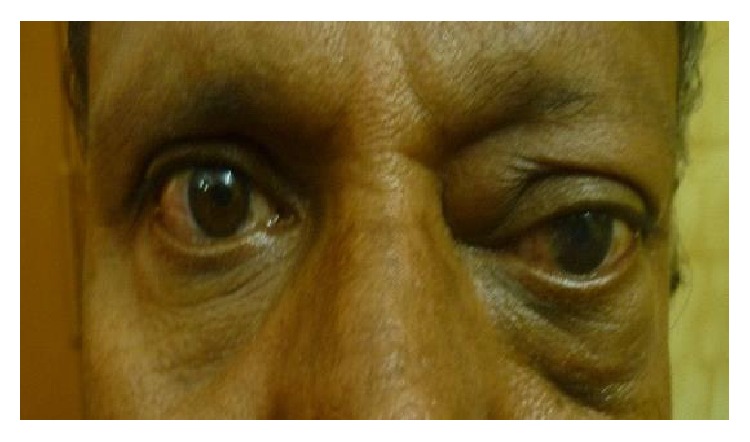
Lymphangioma—eccentric.

The most common etiology seen in our study was inflammation with 8 cases of acute onset (32%), then there were 5 cases of orbital cellulitis (20%), 1 case of mucormycosis (4%), 1 case of orbital apex syndrome (4%), and 1 case of frontal mucocele (4%). Other causes include pseudotumours (2 cases, i.e., 8%) Figure 1(), posttraumatic retrobulbar haemorrhage (1 case, i.e., 4%) Figure 2(), dermoid (1 case, i.e., 4%) Figure 3(), and luxated globe (1 case, i.e., 4%). Malignant tumours were retinoblastoma (1 case, i.e., 4%) Figure 4(), Hodgkin's lymphoma (1 case, i.e., 4%), squamous cell carcinoma (3 cases, i.e., 12%) Figure 5(), and adenocarcinoma of the lacrimal gland (1 case, i.e., 4%); benign tumours were pleomorphic adenoma of the lacrimal gland (1 case, i.e., 4%) Figure 6(), osteoblastoma (1 case, i.e., 4%) Figure 7(), lymphangioma (1 case, i.e., 4%) Figure 8(), acoustic schwannoma (1 case, i.e., 4%), meningioma (1 case, i.e., 4%), and haemangioma (1 case, i.e., 4%) shown in Table 4.

References to ENT, neurosurgery, and oncology were given for any associated conditions. Diagnosis was confirmed clinically and with the help of CT/MRI scans. Scans helped in localising the lesions and gave an idea into the aetiology of the condition and in making a decision in the management of the patient. In tumours, histopathology reports after biopsy wherever possible confirmed the diagnosis.

Inflammations (13) were medically managed with systemic antibiotics and steroids. 12 patients were surgically managed by orbitotomies or orbital exploration, depending on the site, out of which 4 cases were done by the neurosurgeon. Following surgery, 5 underwent radiotherapy and chemotherapy, as shown in Table 5.

No complications were seen in follow-ups.

16 (60%) improved with no disease, 3 (12.5%) were with disease as status quo, 1 (4%) steadily deteriorated, and there was 1 (4%) mortality in the study period.

## 4. Discussion

Orbital pathology usually presents as proptosis. Symptoms reflect the orbital volume increase. Direction indicates the site of lesion [4]. In comparison to other studies where neoplasms are seen to be more common, in our study, inflammations in the orbit were more common and contributed to most of the cases.

There are very few studies available about unilateral proptosis which include all lesions giving rise to unilateral proptosis.

The causes of adult unilateral proptosis may be a retrobulbar haematoma following trauma, inflammatory conditions like orbital cellulitis, an orbital abscess, usually following frontal or ethmoid sinusitis, a pseudotumour of the orbit due to a granuloma of unknown cause, an epidermoid or dermoid cyst, a mixed lacrimal tumour (lacrimal adenoma), or a haemangioma. Malignant tumours include malignant melanoma, carcinoma of the maxillary or ethmoidal sinuses invading the orbit, and meningioma of the sphenoid. Thyroid eye disease which is usually bilateral also can present as a unilateral proptosis in its initial stages [3, 5]. Primary tumours of the orbit are usually mixed tumours of the lacrimal gland and dermoid cysts [2]. Anterior temporal lobe lesions into the orbit can lead to proptosis and blindness [6].

The causes of unilateral proptosis in a child include retinoblastoma in the first 5 years of life and infective orbital cellulitis [7].

The direction of exophthalmos may indicate the likely aetiology and site of lesion [1]. Axial proptosis is seen in tumours arising within the muscle cone like optic nerve glioma. The eyeball is displaced down and/or lateral in diseases of frontal or ethmoid sinuses. Lacrimal gland or temporal fossa tumours have a medial displacement [8].

To evaluate and treat the patient with unilateral proptosis, an ophthalmologist must work closely with the ENT surgeon, neurosurgeon, and radiologist to ensure a successful outcome in each case.

During the past few decades, advances in diagnostic instrumentation and surgical technique have helped to elevate the orbit to an anatomical area of great clinical interest. CT, MRI, and orbital echography have dramatically improved diagnostic accuracy and allowed a more careful therapeutic planning [9, 10]. Orbital surgery has become safer and more precise, and treatment results are significantly enhanced. The operating microscope, specialized orbital instruments, fibre optic illumination, endoscopy, and hypotensive anaesthesia have allowed orbital surgeons to perform complex deep dissections more easily and with fewer complications.

As regards the management, inflammatory cases and benign orbital neoplasms were most amenable to satisfactory treatment, medical or surgical, while malignant primary tumours if detected early could be eradicated with fair chances of success. Surgically, results were more encouraging in cases of retinoblastoma and lacrimal gland tumours than in other highly malignant and infiltrative growths where deep X-ray therapy was the only possible recourse for palliative and temporary improvement.

Proptosis in many cases if undiagnosed and unexplained when deep in the orbit and left to fate is not advisable, so orbital exploration is necessary to clear the worry of the patient as well as the ophthalmologist [11]. Anterior orbitotomy with ethmoidectomy was the most satisfactory approach for anteriorly situated neoplasms, while transfrontal approach by neurosurgeons gave better accessibility for tumours situated medial to the optic nerve or extending intracranially. Palliative decompression of the orbit through this route was found most effective.

## 5. Conclusion

Unilateral proptosis is a multidisciplinary problem and requires collaboration of different specialties [4] of an ophthalmologist along with an otorhinolaryngologist, neurosurgeon, oncologist, and radiotherapist. A thorough ENT examination is mandatory in proptosis [2]. A small number of cases can never go noticed, but in proptosis, however small the bulge, malignancy has to be ruled out. The commonest cause is malignancy in other studies; our study showed more of an inflammatory origin. CT scan was valuable in evaluating a case of proptosis, but histopathological examination provides a definitive diagnosis of the exact aetiology.

## Figures and Tables

**Table 1:** Age distribution.

Age in years	Number of cases	%
0–9	2	8
10–19	0	0
20–29	4	16
30–39	5	20
40–49	5	20
50–59	3	12
>60	6	24

**Table 2:** Type of proptosis.

Axial	15
Eccentric	10

**Table 3:** Presenting symptoms.

Symptoms	No of patients N-25
Proptosis	25
Diminished vision	20
Diplopia	2
Diminished motility	6
Epiphora	2
Headache	24
Chemosis	20
Orbital mass	10
Eye pain	24
Ptosis	5

**Table 4:** Cause of proptosis.

Inflammatory		
(a) Orbital cellulitis	5	20%
(b) Orbital apex syndrome	1	4%
(c) Frontal mucocele	1	4%
(d) Mucormycosis	1	4%
Pleomorphic adenoma of lacrimal gland	1	4%
Pseudotumours	2	8%
Trauma (retrobulbar haemorrhage)	1	4%
Hodgkin's lymphoma	1	4%
Squamous cell carcinoma	3	12%
Lacrimal gland adenocarcinoma	1	4%
Osteoblastoma	1	4%
Meningioma	1	4%
Haemangioma	1	4%
Acoustic schwannoma	1	4%
Retinoblastoma	1	4%
Lymphangioma	1	4%
Dermoid	1	4%
Luxated globe	1	4%

**Table 5:** Treatment modalities.

Treatment modalities	Cases
Medical	13
Surgery	12
Radiation + chemotherapy	05
Referred cases	04
